# Towards Building a Quantitative Proteomics Toolbox in Precision Medicine: A Mini-Review

**DOI:** 10.3389/fphys.2021.723510

**Published:** 2021-08-26

**Authors:** Alejandro Correa Rojo, Dries Heylen, Jan Aerts, Olivier Thas, Jef Hooyberghs, Gökhan Ertaylan, Dirk Valkenborg

**Affiliations:** ^1^Data Science Institute, Interuniversity Institute for Biostatistics and Statistical Bioinformatics (I-BioStat), Hasselt University, Diepenbeek, Belgium; ^2^Flemish Institute for Technological Research (VITO), Mol, Belgium; ^3^Department of Applied Mathematics, Computer Science and Statistics, Faculty of Sciences, Ghent University, Ghent, Belgium; ^4^National Institute for Applied Statistics Research Australia (NIASRA), Wollongong, NSW, Australia; ^5^Theoretical Physics, Data Science Institute, Hasselt University, Diepenbeek, Belgium

**Keywords:** precision medicine, quantitative proteomics, targeted techniques, bioinformatics, biomarker discovery, clinical diagnostics, protein quantitative trait loci

## Abstract

Precision medicine as a framework for disease diagnosis, treatment, and prevention at the molecular level has entered clinical practice. From the start, genetics has been an indispensable tool to understand and stratify the biology of chronic and complex diseases in precision medicine. However, with the advances in biomedical and omics technologies, quantitative proteomics is emerging as a powerful technology complementing genetics. Quantitative proteomics provide insight about the dynamic behaviour of proteins as they represent intermediate phenotypes. They provide direct biological insights into physiological patterns, while genetics accounting for baseline characteristics. Additionally, it opens a wide range of applications in clinical diagnostics, treatment stratification, and drug discovery. In this mini-review, we discuss the current status of quantitative proteomics in precision medicine including the available technologies and common methods to analyze quantitative proteomics data. Furthermore, we highlight the current challenges to put quantitative proteomics into clinical settings and provide a perspective to integrate proteomics data with genomics data for future applications in precision medicine.

## Introduction

Precision medicine aims to stratify patient populations so as to provide targeted and efficient treatments and reduce adverse treatment effects for human health (König et al., 2017). Furthermore, it brings opportunities for the healthcare industry by utilizing novel diagnostics platforms and specialized treatments that combine large-scale data with high-end computational analyses (Flores et al., 2013; Siwy et al., 2019).

The advances of biomedical and molecular technologies reduced per-individual cost of high-throughput technologies, such as next-generation sequencing and targeted proteomics. These advances bring omics sciences as a feasible approach to unravel molecular patterns of disease and wellbeing, and hence put precision medicine into clinical practice (Olivier et al., 2019; Morello et al., 2020). Genomics has been the most used approach given the high amount of available genetic data and its association with traits and chronic diseases, such as cancer (Malone et al., 2020), type II diabetes mellitus (T2D; Scott et al., 2017), and cardiometabolic diseases (Dainis and Ashley, 2018). Still, most genetic studies provide associations between genes and risks for a disease, no direct mechanistic markers are found that explain the disease etiology, expediting the need to associate with other molecular layers and environmental factors (Tam et al., 2019). Despite great scientific and technological developments in recent years, many applications are still at the research-grade level requiring demonstration of clinical validation and usability (Liu et al., 2019).

Proteomics is the next likely candidate to be included in the precision medicine arsenal, for proteins represent intermediate phenotypes. In particular, proteins are products of gene expression and mediate biochemical activities of cells and tissues (Ding et al., 2019). Proteomics approaches could describe disease-related pathways; identify novel biomarkers for diagnostics; detect drug targets; and analyze physiological patterns on the transition for disease (Van Eyk and Snyder, 2018).

More specifically, quantitative proteomics has emerged as an important technique for precision medicine because it provides information about the physiological differences between biological samples based on the protein abundance levels. Thus, quantitative proteomics has relevant applications for the clinical and biomedical field including biomarker and drug discovery (Prasad et al., 2017). For the detection of human proteins, targeted approaches are often used which include targeted mass spectrometry (MS) techniques or affinity-reagent-based platforms. Targeted techniques aim to quantify the abundance of preselected proteins from an individual and thus correlate concentration values with patterns of disease.

Mass spectrometry is the most common technique in proteomics studies and has been widely used to measure proteins in the blood. Recent innovations in MS techniques have brought novel methods to measure human proteins, such as data-independent acquisition (DIA) methods and mass spectrometry imaging (MSI). DIA methods combine the reproducibility of single/parallel/multiple reaction monitoring with the high-throughput discovery aspect of shotgun proteomics while remaining comprehensive (Zhang et al., 2020). Conversely, MSI is transforming pathology allowing to identify precise and quantitative changes of proteins across individuals, disease states, tissues, and time (Ščupáková et al., 2020). Up to date, targeted MS-based blood proteomics have detected more than 17,000 proteins from coding genes in the human proteome (Kim et al., 2014; Adhikari et al., 2020). Yet, implementations for the human blood proteome in clinical settings are limited because targeted MS techniques require multiple sample preparation steps including removal of high-abundance proteins, trypsin digestion, and liquid chromatography (Maes et al., 2015).

Affinity-based methods have been considered as an alternative approach to MS. These are often based on antibodies to target specific proteins in a biological sample and they are considered the gold standard for clinical diagnostics. Classical techniques, such as ELISA, use polyclonal or monoclonal antibodies to capture protein targets (Brennan et al., 2010). However, due to their cross-reactivity, they have poor specificity, and sensitivity for low-abundant proteins in human samples, and they are consequently not suitable for high content, large-scale analyses, or high coverage of human proteins (Ellington et al., 2010).

**Figure 1:**
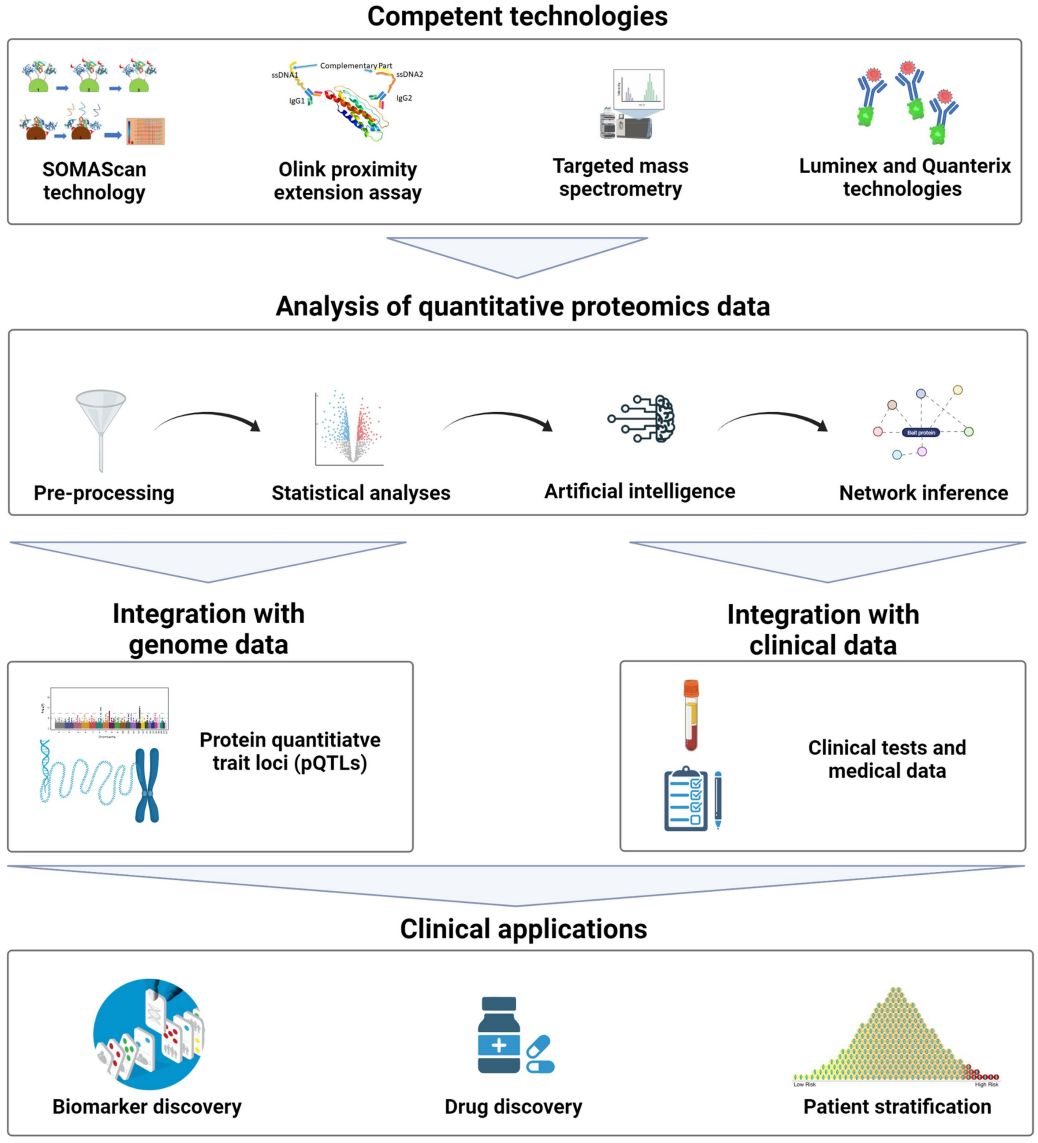
General workflow for quantitative proteomics. The figure describes the different types of targeted technologies, and the common methodologies to analyse quantitative proteomics data. These analyses potentially provide clinical applications in biomarker and drug discovery and patient stratification. Image created with BioRender.

With the advances of multiplexing technologies, immunoassays techniques have been improved for simultaneously measuring multiple proteins with a wide range of concentrations in multiple samples. Compared to targeted MS, multiplexed technologies are high throughput, have high sensitivity for low abundant proteins, and target specific proteins of clinical relevance (Smith and Gerszten, 2017). Therefore, in this mini-review, we will briefly discuss current innovations and applications of quantitative proteomics, based on high-throughput multiplexed technologies, in precision medicine and their current status in the clinic (Figure 1).

## Multiplexed Affinity-Reagent-Based Methods

Multiplexed immunoassay technologies include improved binding reagents to increase affinity and specificity, using multiplexed ELISA arrays (Luminex and Quanterix), antibody labeled nucleotides (Olink), or aptamers (SOMAScan).

Luminex and Quanterix (SIMOA) technologies are based on suspension bed arrays in which captured antibodies are attached to different fluorescent-dyed microparticles. Each colored microparticle represents one assay for a given protein target. Proteins are then measured by flow cytometry analysis (Tait et al., 2009; Rissin et al., 2010). These techniques can quantify up to 50 proteins and process up to 384 samples in batches (Wilson et al., 2016).

Conversely, Olink technology (Olink Proteomics) uses antibodies that are labeled with nucleotides and detect proteins in a sample by proximity extension assay (PEA). Antibodies that are linked with complementary oligonucleotides which upon binding the target protein, the oligonucleotides are hybridized and then extended using a DNA polymerase. The initial concentration of the protein target is measured by the concentration of the generated DNA amplicon, using quantitative PCR (Assarsson et al., 2014). Nowadays, the platform can detect up to 1,162 clinically relevant proteins distributed across 15 protein panels related to cardiometabolic disorders, cell regulation, cardiovascular diseases, immune system, oncology, inflammation, metabolism, and neurology. Additionally, each panel allows multiplexing for 90 samples per batch.

SOMAScan technology (SOMALogic) uses aptamers to achieve high sensitivity and high multiplexing. Aptamers are short oligonucleotides developed by a pool of random sequence oligomers that binds to a target protein. Captured proteins by aptamers are then measured using a DNA microarray (Gold et al., 2010). The current version of this platform can measure more than 7,000 proteins and processes 90 samples per batch.

Compared to MS techniques, multiplexed affinity-reagent-based methods achieve high coverage, high sensitivity for several low abundances, high specificity for target proteins, and good reproducibility (low intra-assay coefficient variation; Smith and Gerszten, 2017; Petrera et al., 2021). However, they have several limitations which include as: no detection of proteins that are not targeted by the assay (comprehensiveness); binding affinity differences across proteins or non-specific binding for variant proteins (quantitative accuracy); and no distinction between posttranslational modified proteins and isoforms (specificity; Yeh et al., 2017; Raffield et al., 2020). For SOMAScan and Olink, Pietzner et al. (2021) showed that factors of technical variability can be introduced by target proteins with transmembrane domain, glycosylation effects, or protein-altering variants (Pietzner et al., 2021). Still, implementations of multiplexed platforms into clinical settings are relatively new, given that more research and verification are still needed to validate these as clinical-grade technologies (Williams et al., 2019). For more information about the recent technical validation of these platforms, we encourage the readers to review the work of Petrera et al. (2021) and Pietzner et al. (2021).

Nevertheless, multiplexed affinity-based methods are now been used for large-population analyses to link proteomics data with genomic data. Affinity-based assays provide a direct link between protein levels and genetic variants which can unravel causes of complex traits and detect biological effects on the protein layer. We provide a summarized table with the current large-population cohorts using these techniques (Table 1).

**Table 1:** Summary of large-scale population cohorts with quantitative proteomics data.

Study population	Research topic	Number of samples	Number of proteins measured	Type of sample	Number of associated proteins with genetic effects	Platform	Reference
KORA and QMDiab study	Association between genetic risk and plasma proteins for diseases	1,335	1,124	Plasma	284	SOMAScan	Suhre et al., 2017
INTERVAL study	Association study between genetic variants and the human plasma proteome	3,301	3,622	Plasma	1,478	SOMAScan Olink Proteomics	Sun et al., 2018
AGES Reykjavik study	Epidemiologic study to examine risk factors and gene–environment interactions for disease and disability in old age	5,457	4,137	Serum	2,148	SOMAScan	Emilsson et al., 2018
LifeLines Dutch population cohort	Multi-generational cohort study to study the etiology of several diseases	1,264	92	Plasma/Serum	74	Olink Proteomics	LifeLines cohort study et al., 2018
SCALLOP consortium	Collaborative framework across multiple studies to map pQTLs and analyze biomarker proteins on the Olink proteomics platform	30,931	90	Plasma/Serum	85^1^	Olink Proteomics	Folkersen et al., 2020
Latin Americans ORIGIN study	Genetic study on the human serum proteome for novel biomarkers in cardiovascular diseases	2,216	237	Serum	23	Luminex immunoassay	Sjaarda et al., 2020

1 *The number of detected proteins showed in the table represents the first published results of the SCALLOP consortium. More population cohorts are being added to the consortium which currently comprises data from 71,232 samples*.

## Analysis of Quantitative Proteomics Data

The analysis of quantitative proteomics data is quite challenging. Depending on the targeted technology used, experimental design, and the type of research question being addressed, specific computational workflows are needed. Bioinformatics has provided a wide range of methods, not only to analyze large-scale proteomics data but also to integrate it with other types of omics data for clinical research. However, standardized workflows are needed to successfully put quantitative proteomics analyses into clinical practice (Martens, 2013). In this section, we review the common and promising methods for analyzing proteomics data based on large-scale studies.

### Data Pre-processing

In omics data analysis, bias refers to systematic features of the data that can be attributed to experimental and/or technical factors that are related to sample preparation, the platform runs, data acquisition, etc. Normalization is the process that aims to correct such biases (Välikangas et al., 2016). In comparison with targeted MS techniques, normalization in multiplex affinity-reagent-based methods is relatively straightforward. The main assumption on these techniques is that protein levels are measured based on targeted antigen/antibody affinity-binding. This implies that abundance levels are not influenced by factors that cause protein isoforms, such as, posttranslational modifications or spliced variants. However, as mentioned before, recent studies have shown biological variations that interfere with the analysis of the data which require further research on pre-processing methods. Nevertheless, we discuss the current approaches used for quantitative proteomics data.

Before normalization, traditional quantitative proteomics data must be transformed to adjust for the effect of protein levels and detect changes in abundances between samples (Quackenbush, 2002). Several methods exist but the most frequently used is the log_2_ transformation because it allows easy interpretation of fold change in protein levels (Karpievitch et al., 2012). After transformation, normalization is applied. The most common methods derived from MS techniques or microdata array methodologies include global and quantile normalization (Bolstad et al., 2003; Chawade et al., 2014), regression models (Callister et al., 2007), and constrained optimization, such as CONSTANd (Maes et al., 2016). However, for Olink and SOMAScan, the pre-processing starts from normalization as the manufacturers provide their normalization guidelines. For Olink, data are normalized based on normalized protein expression values (NPX) (Sun et al., 2018; Zhong et al., 2021) while for SOMAScan, data are normalized by estimating relative fluorescence intensities (RFUs; Candia et al., 2017).

Batch effects are also an important consideration in data pre-processing. Although normalization methods aim to correct for these effects simultaneously, some sources of variations are resistant to these approaches. For large proteomics datasets, empirical Bayes methods, such as ComBat (Johnson et al., 2007; Leek et al., 2012), have been used to adjust for known batch effects (Kim et al., 2018; Kalla et al., 2021).

Despite the availability of multiple pre-processing methods for quantitative proteomics data, the main limitation is the lack of methodologies to compare protein levels between multiple cohorts. The application of the previously mentioned methods is not yet fully studied and transparently communicated. Validation of these methods for affinity-based techniques is necessary to compare data from multiple targeted platforms and obtain reproducible results (Rausch et al., 2016).

### Statistical and Enrichment Analyses

Traditional statistical analyses compare protein levels between study groups or conditions and detect which proteins are significantly differentially expressed. This is commonly done by performing two-sample *t*-tests between protein abundances or an ANOVA when two or more conditions are to be compared (Kammers et al., 2015). For more robust and accurate results, Linear Models for Microarray Data (LIMMA) are used (Ritchie et al., 2015).

For large-scale proteomics analyses, multiple hypotheses are being tested which is necessary to control for false positives. Statistical estimates, such as false discovery rate and the Benjamini-Hochberg procedure (BH), are used to obtain true biological results (Aggarwal and Yadav, 2016; Korthauer et al., 2019).

In addition to the previously mentioned methods, Olink Proteomics offers an open-source toolbox, OlinkAnalyze, to pre-process and do quick analyses for Olink’s data.^1^ Conversely, SOMALogic also provides a platform for the pre-processing and analysis of aptamer-based proteomics data.^2^

Results from statistical analyses do not yet provide the biological context of differentially expressed proteins. To understand the functional features and effects of the detected proteins, an enrichment analysis must be performed. This helps to generate hypotheses on the systemic response of the proteome, revealing and understanding the biological processes that underlie the quantitative profiles of the proteins. Methods include simple classification of proteins using large public databases, such as UniProt (The UniProt Consortium et al., 2021) and Ensembl (Howe et al., 2021), and Gene Ontology (GO) analyses from resources, such as AmiGO database (Carbon et al., 2009); EggNOG (Jensen et al., 2007); and MetaCore^™^.

### Artificial Intelligence-Based Methods

Artificial intelligence-based methods can extend traditional statistical analyses by extracting informative features and building models that can predict or describe relevant outcomes. Using supervised and unsupervised techniques, a variety of models include Random Forest, support vector machines (SVMs), Artificial Neural Network, regression models, and K-means clustering (Chen et al., 2020). In quantitative proteomics, based on multiplexed affinity-reagent-based methods, these techniques have been used to predict disease signatures or clinical outcomes. Suvarna et al. (2021) identified protein classifiers of patients with non-severe and severe COVID-19, by using SVMs models (Suvarna et al., 2021). Hewitson et al. (2021) used Random Forest and logistic regression models to classify proteins in blood as potential biomarkers in autism spectrum disorder (Hewitson et al., 2021).

### Network Inference

Mapping interactions and associations between different proteins allow presenting proteomics data as networks. These interactions reflect molecular entities as building blocks of any type of biological process, especially signaling, regulation, and biochemical interactions. Two distinct strategies of network inference are possible. Validated pathways and mechanisms can be consulted in resources, such as KEGG (Kanehisa et al., 2016), ENCODE (The ENCODE Project Consortium, 2012), PathVisio (Kutmon et al., 2015), MetaCore^™^, WikiPathways (Slenter et al., 2018), Reactome (Jassal et al., 2019), BioGrid (Stark, 2006), STRING (Jensen et al., 2009), and iPathwayGuide^™^. Such knowledge-based approach can guide integrative analyses by making use of established information from validated experiments, databases, and scientific literature.

In a more data-driven approach, statistical or machine learning methods can be used for inferring relationships, correlating between proteins and/or other molecules, and exploring novel interactions. Common methods include weighted gene co-expression network analysis, Gaussian graphical models, Bayesian networks, and Markov Chain Monte Carlo (MCMC; Mohammadi and Wit, 2015; Hawe et al., 2019).

## Integration with Genomic Data

Genomics have always been the key technology in personalized medicine. Genome-wide association studies (GWAS) have been used to test millions of genetic variants across many individuals to identify genotype–phenotype associations. Overall, more than 50,000 associations have been reported between genetic variants, common diseases, and traits (Loos, 2020). However, GWAS has not been able to bridge the gap between genotype and phenotype because most of the identified associations only explain a small fraction of heritability and do not provide causality between genetic variants and traits.

Quantitative proteomics can extend GWAS toward proteome-wide association studies (PWAS) by studying protein quantitative trait loci (pQTLs). pQTLs refer to associations between genetic variants and protein abundance levels which can be *cis*-pQTLs or *trans*-pQTLs (Suhre et al., 2021). *Cis*-pQTLs specify variants that are likely to have a direct effect on the observed protein levels at that locus, whereas *trans*-pQTLs specify a variant distant to the protein-coding gene or on another chromosome that could indicate an indirect link (Molendijk and Parker, 2021).

In the context of precision medicine, several studies have successfully described phenotypic features of complex diseases using PWAS. Wingo et al. (2021) integrated 376 human brain proteomes with GWAS data from 455,528 individuals in which 13 coding genes were found causal for protein levels as well to be correlated with Alzheimer’s disease, neuroticism, and Parkinson disease (Wingo et al., 2021). Zaghlool et al. (2021) studied the association between 1,000 plasma proteins and body mass index over 4,600 participants where 21 proteins in pathways of adiposity were found to be causal drivers in obesity-associated pathologies (Zaghlool et al., 2021).

## Clinical Applications

The applications for quantitative proteomics in precision medicine are numerous. Proteomics promises to contribute to the stratification of treatment options for patients. It can provide robust support for biomarker discovery and drug development. Additionally, it can be integrated with genetic data to support genetic risk scores for complex diseases. Before these potential applications of quantitative proteomics can be realized, an important consideration is that proteomics data may reveal personal data. Hence, ethical, privacy, and data sharing frameworks are needed to allow secured research in precision medicine (Boonen et al., 2019). Below, we highlight three promising applications of quantitative proteomics in the clinic.

### Diagnostics, Biomarker Discovery, and Surrogate End-Points

In general, most proteomics studies in the clinic are aimed at the identification of biomarkers that are specific for the diagnosis of disease or associated with disease severity. Recent studies have identified potential biomarkers for different types of disease. Franzén et al. (2021) identified 33 protein biomarkers of non-small-cell lung cancer related to different stages of disease severity (Franzén et al., 2021). Sonnenschein et al. (2021) identified c-KIT as a novel biomarker from serum proteins to distinguish between patients with hypertrophic cardiomyopathy and healthy subjects (Sonnenschein et al., 2021).

### Pharmacoproteomics

Integration of genomic data in large-scale proteomics studies is now providing novel methodologies for drug target identification. With the ongoing research on pQTLs, recent GWAS and PWAS have identified potential drug targets for several diseases. From one UK Biobank study, Bretherick et al. (2020) detected 38 proteins with pQTL effects in inflammatory bowel disease, coronary artery disease, and schizophrenia. From these proteins, 1,319 compounds were associated as potential therapeutic agents (Bretherick et al., 2020).

### Polygenic Risk Scores

Polygenic risk scores (PRSs) are a novel approach to integrate individual genetic data into clinical settings. These scores aggregate the effect of multiple risk variants to assess the individual genetic predisposition for a given disease (Lewis and Vassos, 2020). Proteomics analyses can be embedded in PRSs, not only for novel biomarkers but also to assess the causes and prognosis of disease. Few studies for coronary artery disease and T2D have successfully integrated PRSs with protein levels which have provided novel associations between gene and protein levels as well as individual risk profiles for disease progression (Benson et al., 2018; Gudmundsdottir et al., 2020).

## Conclusion

Quantitative proteomics is emerging as a powerful technology for precision medicine. For decades, MS has been the standard for quantitative proteomics for researchers, but new alternatives in affinity-reagent-based assays allow for high-throughput screening of proteins. Recent innovations provide tools for clinicians to medical applications, including in diagnostics, stratification, and treatment of diseases. However, substantial work is required for the validation of technologies, standardization of data analyses, and integration of proteomics with other molecular and phenotypic level data. Despite these challenges, recent progress is promising for the emerging quantitative proteomics toolbox to be used in clinical settings.

## Author Contributions

AR, DH, GE, and DV have equally contributed to the conceiving of the manuscript idea. AR and DH have drafted the manuscript with support from GE and DV. JA, JH, and OT have provided critical comments on the draft manuscript. All authors read and approved the final manuscript.

## Conflict of Interest

The authors declare that the research was conducted in the absence of any commercial or financial relationships that could be construed as a potential conflict of interest.

## Publisher’s Note

All claims expressed in this article are solely those of the authors and do not necessarily represent those of their affiliated organizations, or those of the publisher, the editors and the reviewers. Any product that may be evaluated in this article, or claim that may be made by its manufacturer, is not guaranteed or endorsed by the publisher.

## Footnotes

^1^ https://github.com/Olink-Proteomics/OlinkRPackage

^2^ https://github.com/SomaLogic/SomaLogic-Data
